# A Study of Sr Sorption Behavior in Claystone from a Candidate High-Level Radioactive Waste Geological Disposal Site under the Action of FeOOH Colloids

**DOI:** 10.3390/ijerph19169970

**Published:** 2022-08-12

**Authors:** Jinsheng Wang, Weihai Cai, Rui Zuo, Can Du

**Affiliations:** 1 College of Water Sciences, Beijing Normal University, Beijing 100875, China; 2 Engineering Research Center of Groundwater Pollution Control and Remediation, Ministry of Education, Beijing 100875, China; 3 Development and Research Center, China Geological Survey, Beijing 100037, China

**Keywords:** FeOOH colloid, claystone, strontium, sorption, geological disposal site

## Abstract

Colloids have a significant influence on the migration of nuclides in claystone, which is an important geological barrier. The sorption of strontium on claystone in the presence of FeOOH colloids was investigated in samples from the Suhongtu site, a candidate high-level radioactive waste disposal site in China. The effects of colloid amount, solid content, and pH were investigated by batch tests, and the sorption reaction mechanism was analyzed by kinetic modeling and microscopic characterization techniques. The results indicate that the sorption of Sr by claystone increased with the solids content, and the claystone had a stronger Sr sorption capacity under alkaline conditions. The Sr sorption kinetics were best described by the pseudo-first-order and pseudo-second-order models, which revealed that the progress is affected by physical diffusion and chemical sorption. Furthermore, the microscopic characterization results demonstrate that cation exchange reactions and surface complex reactions are the main sorption mechanisms for Sr sorption on claystone. Ca and Mg plasmas in claystone minerals can have cation replacement reactions with Sr, and functional groups such as -OH and [CO_3_]^2−^ can have complexation reactions with Sr to adsorb Sr on the surface of the claystone. Additionally, the presence of the FeOOH colloid inhibited the sorption effect of claystone slightly. The FeOOH colloid could occupy sorption sites on the claystone surface, which reduces the activity of the functional groups and inhibits the sorption of Sr on claystone.

## 1. Introduction

Nuclear energy has greatly increased global power generation capacity and will play an increasingly important role in the world’s energy system [1]. However, the proper disposal of radioactive waste generated by the development and utilization of nuclear energy cannot be ignored [2,3]. The geological disposal method is currently recognized as the safest and most effective method of nuclear waste disposal in the world [4]. The sorption characteristics of nuclides on natural barrier media such as geological bodies are some of the important criteria in evaluating the safety of a repository [5]. Claystone is an important disposal repository envelope [6]; therefore, it is critical to study its sorption behavior for nuclides. Claystone is widely distributed in the Suhongtu area of Inner Mongolia, which is a candidate site for a high-level radioactive waste (HLW) repository in China [7].

^90^Sr is a typical component in radioactive nuclear waste and is characterized by a long half-life. It is biologically and chemically toxic, easily migrates in environmental media, and accumulates through biological chains [8,9]. The chemical properties of Sr are very similar to those of Ca and Mg. Once it enters the human body through the food chain, Sr can easily replace Ca in human bones, causing bone hematoma, leukemia, etc., and endangering life [10,11]. Therefore, it is important to study the sorption behavior of environmental media on the typical nuclide Sr for the prevention and control of Sr contamination.

To date, other scholars in the field have carried out many studies on the sorption of Sr by different geological disposal media, such as kaolinite [12], montmorillonite [13], granite [14,15], and bentonite [16]. The existing research methods are mainly mathematical models, batch adsorption tests, microscopic characterization technology, and geochemical simulation, etc. Some progress has been achieved in the study of the adsorption mechanism of Sr on nuclear waste geological treatment medium by these methods. For example, sorption kinetics studies have shown that the sorption of Sr in geological disposal media is mainly physical and chemical sorption [17]. Sr can also react with clinoptilolite in complex reactions and ion exchange reactions, and the sorption process is affected by intraparticle and surface film diffusion [18]. In addition, batch experiments show that the sorption behavior of Sr can be influenced by many factors, such as temperature [19], pH [20], colloids [21,22], solid amount [23,24], initial concentration [25], and ionic strength [26]. From the above research, it can be recognized that the adsorption behavior of Sr in the claystone of geological disposal repositories is complex and affected by many factors. However, there is a lack of effective research tools, and further research about the interaction mechanisms between claystone and Sr is needed.

Colloids are defined as molecular polymers or macromolecules with particle sizes ranging from 1 nm to 1 μm and are divided into organic colloids and inorganic colloids, which widely exist in geological media and groundwater [27,28]. There are many functional groups in colloids, which can form complexes with metals and radionuclides, affecting radionuclide sorption and migration in geological media [29]. Numerous studies have been conducted on the effect of colloids on the sorption of adsorbed nuclides in media. Zuo et al. [30] found that silicate colloids can promote the sorption of Sr in the groundwater environment, and the amount of sorption is related to the amount of the colloids, the amount of sorption media, and the pH. Luo et al. [31] showed that soil colloids rely mainly on functional groups such as reactive carboxylates and alcohol groups to adsorb vanadium (V) and that high ionic strength reduces their sorption capacity. Other colloids, such as bentonite colloids, humic acid colloids, and kaolin colloids, can also have an influential impact on sorption [14,22,32]. Goethite is a typical mineral in geological barrier media and often exists as a colloid in groundwater environments [33]. Previous studies have found that the adsorption kinetics of Sr on goethite conform to a pseudo-second-order kinetic model and isothermal adsorption conforms to the Langmuir model. -OH plays an important role in the sorption process, and inner-sphere coordination is the main sorption mechanism [34,35]. Nevertheless, understanding the influence of FeOOH colloids on the sorption of Sr by geological media is still in the preliminary exploration stage.

This study compares the sorption behavior of Sr on claystone (CT) and claystone with the addition of FeOOH colloids (CF) and explores the influence of colloid addition, the amount of the sorption medium, and the pH on sorption. The sorption reaction mechanism was explored by using sorption kinetics and microscopic characterization techniques. This work is expected to provide a theoretical basis for the selection of high-level radioactive waste geological disposal sites and the control of nuclide pollution, especially ^90^Sr, at the Suhongtu site.

## 2. Materials and Methods

### 2.1. Materials

**Figure 1:**
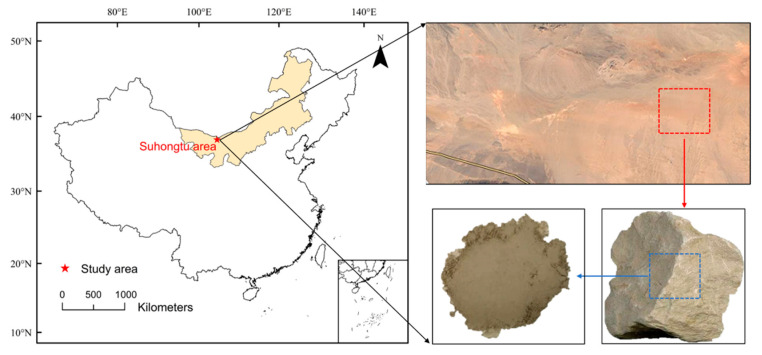
Claystone enclosures in the Suhongtu area.

In this study, the claystone that is widely found in the Suhongtu area was selected as the sorption medium. As shown in Figure 1, the Suhongtu area is located in the western part of the Inner Mongolia Plateau. Claystone is widely distributed and has excellent properties, with low permeability, strong adsorption capacity and strong plasticity. The Suhongtu area is an alternative site for high-level radioactive waste in China.

The results of the tests on the mineral composition of the claystone from the Suhongtu area showed that the content of Fe_2_O_3_ was approximately 5.36%; therefore, the FeOOH colloid was used as the main colloid for the sorption experiments in this study. FeOOH was prepared in the laboratory, and the preparation process is described in Supplementary S1.1 (Materials and sample pretreatment).

XRF was used to analyze the composition and chemical state of the substances contained in the claystone samples, and showed that the main elements in the claystone are Ca, Si and Mg, accounting for 34.62%, 26.72%, and 11.69% of the total elements, respectively, and the main oxides are SiO_2_ (36.59%), CaO (24.73%), MgO (14.11%) and Al_2_O_3_ (10.00%). The test results are shown in Supplementary Table S1 (Types and concentration of oxides in claystone samples determined by XRF).

### 2.2. Experiment Methods

According to the element type and content measured by XRF, the content of Sr in the experimental sample was 434 ± 0.1 μg/g. To maximize the effect of claystone mineral dissolution on the Sr concentration in the solution system, the concentration of Sr in the static sorption experiment was set to 20 mg/L. In addition, the pH of groundwater in the Suhongtu area under actual conditions is approximately 6–9, and the effect of the groundwater composition is not significant at temperatures from 10 to 70 °C. Therefore, the experimental conditions in this study were set at pH = 7 ± 0.2 and a temperature of 25 °C.

During the experiment, two different solutions were used: ① Sr solution (20 mg/L) ② Sr solution (20 mg/L) + FeOOH colloid (10 mg/L). The solution volume was set to 250 mL, the temperature was 25 °C, and the sorption time was 4320 min. After the experiment, one portion of the sample solution (S1) was retained, and one portion of the sample (S2) was passed through a 0.10 μm filter membrane. The concentration of Sr in the adsorption solution was then determined by inductively coupled plasma atomic emission spectrometry (ICP-AES).

The main influencing factors considered in this study were the amount of added colloid, the amount of the sorption medium, and the pH. While changing each factor considered during the experiment, the other experimental conditions were kept the same, all experiments were conducted in triplicate, and the experimental results were averaged to exclude the influence of accidental errors. The experimental conditions are shown in Supplementary Table S2 (Controlled and varied factors of the experiments), and the specific experimental procedure is shown in S1.2 (Methods).

### 2.3. Data Analysis Methods

In this study, the sorption effect of claystone on Sr was estimated by the sorption quantity (*Q_t_*) and the sorption rate (*R*), and the sorption process that occurred was analyzed using the relationship between the mass concentration of the adsorbent and time. The main sorption kinetic models used were the pseudo-first-order model [36], the pseudo-second-order model [37], the Elovich equation [38], and the intraparticle diffusion equation [39].

Sorption quantity:

(1) $$Q_{t}=\frac{\left(C_{0}-C_{t}\right)V}{m}$$

Sorption percentage:

(2) $$R=\frac{C_{0}-C_{t}}{C_{0}}\times 100\%$$

where *Q_t_* is the amount adsorbed (mg·g^−1^), *C*_0_ and *C_t_* are the initial and equilibrium liquid-phase metal concentrations (mg·L^−1^), m is the mass of the adsorbent (g), and *V* is the volume of the solution (L).

Pseudo-first-order (PFO):

(3) $$q_{t}=q_{e}\left(1-e^{-k_{f}t}\right)$$

Pseudo-second-order (PSO):

(4) $$q_{t}=\frac{k_{s}q_{e}^{2}t}{1+k_{s}q_{e}t}$$

Elovich equation:

(5) $$q_{t}=\frac{1}{\beta }\ln\left(\alpha \beta \right)+\frac{1}{\beta }\ln\left(t\right)$$

Intraparticle Diffusion:

(6) $$q_{t}=k_{p}\sqrt{t}+C$$

where *q_e_* and *q_t_* are the amounts of Sr (mg∙g^−1^) adsorbed at equilibrium and at time *t* (min), respectively; *k_f_* and *k_s_* are the equilibrium rate constants; *α* is the initial adsorption rate (mg∙ g^−1^∙min^−1^); *β* is desorption constant (g∙mg^−1^); *k_p_* is the rate constant (mg∙g^−1^∙min^−1/2^); and *C* is a constant (mg·g^−1^) controlled by the thickness of the boundary layer. The agreement between the experimental data and the model calculated values is expressed by the determination coefficient (*R*^2^). The relatively high *R*^2^ value indicated that the model successfully follows the kinetics of Sr sorption.

### 2.4. Characterization

The microcosmic surface morphology of the samples was observed using a scanning electron microscope (Hitachi SU8010, Hitachi, Tokyo, Japan). The functional groups of the samples were analyzed using a Fourier transform infrared spectrometer (NEXUS 670, Thermo Fisher Scientific, Waltham, MA, USA). The spectra were collected using KBr powder as a matrix in the range of 450–4000 cm^−1^. X-ray powder diffraction (XRD) patterns were recorded on a DRON-4-07 X-ray diffractometer (Bourevestnik, St. Petersburg, Russia) in the range of 10° < 2θ < 80° using Cu Kα irradiation. XPS analysis was performed using an ESCALAB 250XI X-ray photoelectron spectrometer (Thermo Fisher Scientific, Waltham, MA, USA), selecting Al Kaas as the X-ray source.

## 3. Results and Discussion

### 3.1. Factors Influencing Sr Sorption onto the Sorbent

#### 3.1.1. Effect of Colloid Amount

**Figure 2:**
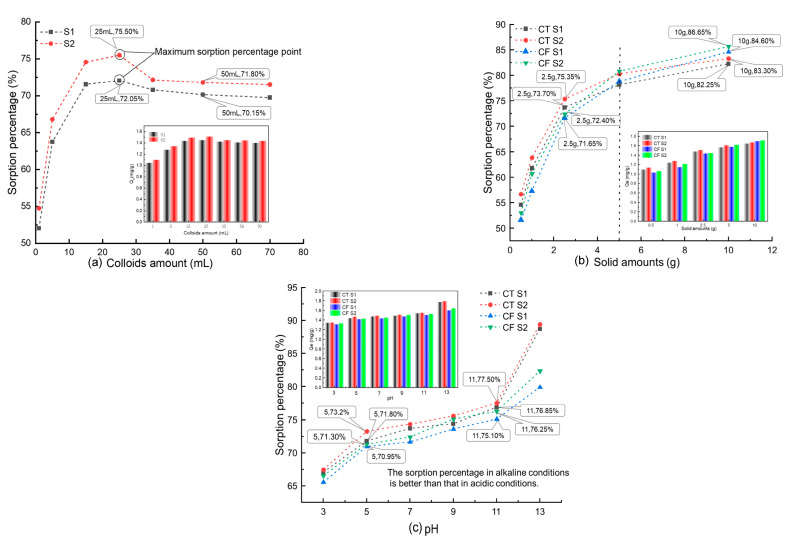
Influence of different parameters on the sorption of Sr by CT and CF: (**a**) colloid amount, C_[Sr]initial_ = 20 mg/L, solid content = 2.5 g, pH = 7.0, t = 4320 min; (**b**) solid content, C_[Sr]initial_ = 20 mg/L, colloid amount = 35.7 mL, solid content = 2.5 g, pH = 7.0, t = 4320 min; (**c**) pH, C_[Sr]initial_ = 20 mg/L, colloid amount = 35.7 mL, solid content = 2.5 g, t = 4320 min.

The effect of the colloid amount (1, 5, 15, 25, 35, 50, and 70 mL) at an initial concentration of 20 mg L^−1^ Sr and 2.5 g CT is shown in Figure 2a. The data showed that the sorption percentage of Sr by CT increased with increased colloid addition and reached the maximum when the colloid addition was 25 mL, which was 72.05% for the unfiltered sample stock solution (S1) and 75.50% for the solution filtered by a 0.10 μm filter membrane (S2). The sorption percentages of Sr by CT showed a gradual decrease and then stabilized when the colloid addition was greater than 25 mL.

Similar results can be found in the sorption of Sr by the soil under the action of humic acid colloids [22]. When the addition of colloids is small (<25 mL), as the colloid concentration increases, the adsorption sites in the system increase, and therefore, the adsorption capacity for Sr [25]. Thus, the sorption percentage of Sr by CT increases with the increase in colloid concentration. Then the addition of colloids continues to increase, the content of free colloids and other ions, such as Fe^3+^ and Fe^2+^, in the system then increases and competes with Sr, and the sorption percentage decreases because of this competition with Sr.

In addition, according to Figure 2a, the sorption percentage of S2 is always greater than that of S1. As mentioned above, S1 is the sample solution, and S2 is filtered by a 0.1 μm filter membrane. Based on the range of colloid particle sizes under natural conditions, the main components of S1 include solid-phase media particles, colloid-mineral agglomerates, Sr-colloid systems, colloid particles, and various ions formed by the dissolution of media minerals. The colloidal material in S2 was filtered, and the Sr loaded on it was filtered with it, so the sorption percentage of Sr in S2 was higher. The experimental results confirm the influence of the FeOOH colloids on the sorption process.

#### 3.1.2. Effect of Solid Content

Figure 2b shows the sorption curves and amounts of Sr (20 mg L^−1^) on CT and CF at different solid contents. In the CT-Sr solution system, the sorption rate increased from 54.60% to 82.25% (S1) and 56.70% to 84.30% (S2) when the claystone mass increased from 0.50 g to 10.00 g. When the claystone mass was less than 2.50 g, the growth rate of the sorption percentage of Sr by claystone was higher. However, when the mass of claystone was more than 2.50 g, the growth rate decreased with the increase in the mass of claystone. The trend of the sorption percentages with the amount of the sorption medium was consistent with CT after the addition of FeOOH colloids to the system.

The results of this study are analogous to the sorption characteristics of Sr on bentonite and granite [40,41]. As the adsorbent mass increases, the adsorbent surface area and sorption sites increase [42]; thus, more Sr is adsorbed on the claystone surface. Notably, Figure 2b shows that when the amount of the adsorbent medium is less than 5 g, the FeOOH colloid shows an inhibitory effect on Sr sorption by claystone, and when the amount of adsorbent medium is greater than 5 g, the FeOOH colloid shows a facilitating effect on Sr sorption by claystone. On the one hand, the FeOOH colloid can compete with Sr to capture the adsorption sites on the surface of the claystone, and on the other hand, the colloid can adsorb Sr in solution to form pseudo-colloids. From Figure 2b, it can be observed that when the claystone mass is small (less than 5 g), the adsorption sites are limited and the colloids play an inhibitory role. With the increase of claystone mass, the surface adsorption sites increase and more pseudo-colloids adsorb on the surface of the claystone, which shows a facilitative effect.

#### 3.1.3. Effect of the pH

Figure 2c shows the sorption curves and amounts of Sr (20 mg L^−1^) on CT and CF at different pH values. The results show that strong acidic and alkali conditions have an obvious influence on the sorption of Sr by CT, and the sorption performance of Sr by CT is stronger under alkaline conditions. In the CT-Sr system, the sorption percentage increased from 67.05% to 88.7% for S1 and 67.45% to 89.4% for S2 when the pH increased from 3 to 13. In the CF-Sr system, the variation in the sorption percentages was consistent with that in the CT-Sr system, but the sorption percentages were lower.

This sorption behavior is consistent with the sorption of Sr on Kula volcanic rock [43] and uranium on salt rock oxide composites [44] and humic acid [45]. Under acidic conditions, the electrostatic force acting on the surface of claystone decreases. At the same time, H^+^ in the sorption system competes with Sr^2+^ for the sorption sites on the surface of claystone, thereby reducing its sorption efficiency for Sr. With increasing pH, the OH^−^ in the solution increases, the competitive sorption of H^+^ weakens, and the activity of -OH and other functional groups on the surface of the claystone in the alkaline environment increases. These functional groups react with Sr to form Sr(OH)_2_ and SrCO_3_ at higher pH [41,46], which increases the percent sorption of Sr.

According to the experimental results, the FeOOH colloid had an obvious inhibitory effect on the sorption of Sr by claystone in a strongly alkaline environment. This is because in a strong alkali environment, the OH^−^ in the solution can combine with free Fe^3+^ to form FeOOH that attaches to the claystone surface, which reduces the sorption sites on the claystone surface and inhibits its sorption of Sr.

### 3.2. Interaction Mechanism

#### 3.2.1. Sorption Kinetics Analysis

**Figure 3:**
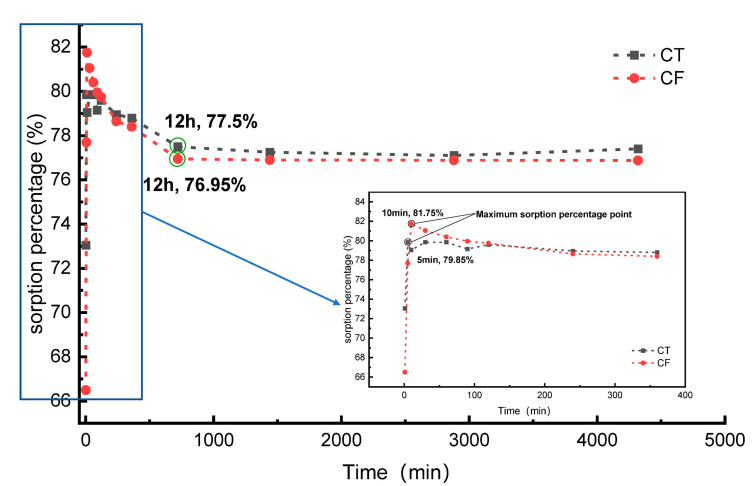
Sorption percentage for the sorption of Sr by CT and CF. C_[Sr]initial_ = 20 mg/L, colloid amount = 35.7 mL, solid content = 2.5 g, pH = 7.0.

Figure 3 shows the sorption percentages of Sr (20 mg/L) onto CT and CF. The sorption percentage of Sr by CT reached a maximum value of 79.85% at 5 min after the beginning of the experiment. Then, the sorption percentage decreased slightly and gradually stabilized, and the system reached equilibrium at 12 h. The sorption rate reached approximately 77.50% at the equilibrium stage. The percent sorption of Sr by CF reached a maximum value of 81.75% at 10 min after the start of the experiment. The system reached equilibrium at 12 h, and the sorption rate at the equilibrium stage was approximately 76.95%. Compared with the case without colloidal action, the FeOOH colloid had a slight inhibitory effect on the process of Sr sorption by claystone.

Similar experimental results can be observed in the sorption behavior of porous media [25], granite [14], and dolomite [47] for Sr with sorption times of 10 h, 24 h, and 2 h, respectively. The sorption media used in this experiment are somewhat different from the above materials, and thus, the sorption reaction processes are different and the sorption equilibrium times vary.

To further investigate the sorption mechanism and sorption behavior of claystone on Sr, four models, the pseudo-first-order model, pseudo-second-order model, Elovich equation, and intraparticle diffusion equation, were fitted to the experimental data within 4320 min from the beginning to the end of the experiment in this study, and the fitting results are shown in Table 1.

The fitting results show that the sorption behavior of Sr on CT follows the pseudo-first-order model (PFO) and pseudo-second-order model (PSO) sorption kinetic models with determination coefficients *R*^2^ > 0.99. Combined with the characteristics of the sorption percentage curve with time, these results reveal that the sorption of Sr by claystone can be divided into two stages. At the early stage of the experiment (≤10 min), sorption sites on the claystone surface were extremely numerous; thus, the sorption rate was fast, and a large amount of Sr was adsorbed onto the claystone surface in a short time. The main driving force at this stage was diffusion, and the process was mainly physical sorption [48]. With increasing sorption time, the sorption sites on the claystone surface decreased, and thus, the sorption rate decreased. It is worth noting that the adsorption percentage decreased by 2.35% at this stage. A similar phenomenon can be found in the study of Zuo et al. [49]. The reason for this phenomenon may be that claystone have agglomerated, resulting in the decrease of adsorption sites and the desorption phenomenon [50]. At this stage, Sr and the claystone surfaces undergo ion exchange and complexation reactions, which are mainly based on chemisorption [51]. The equilibrium sorption amounts calculated by the PFO and PSO models were in general agreement (1.56 mg·g^−1^), and the relative error between the experimental results (1.54 mg·g^−1^) was small, indicating a high degree of confidence in the fitted results. In addition, the determination coefficient *R^2^* was <0.05 for the intraparticle diffusion model, so the sorption behavior of the claystone on Sr does not follow the intraparticle diffusion model; that is, the rate-limiting step of sorption on Sr in the claystone is not an intraparticle diffusion effect.

The sorption process of Sr by CF was similar to that described above, following PFO and PSO with a combination of physical and chemical sorption. However, the equilibrium sorption of Sr by CF was smaller than that of CT in both the experimental results and the fitted results. Furthermore, according to Table 1, the rate constants of CF (1.95 min^−1^, 4.40 g∙mg^−1^∙min^−1^) are all smaller than those of CT (2.745 min^−1^, 11.12 g∙mg^−1^∙min^−1^). This indicates that the FeOOH colloids had an inhibitory effect on Sr sorption by claystone, which may be because the FeOOH colloids occupied the sorption sites on the surface of claystone, reducing its sorption capacity.

#### 3.2.2. Microstructure Analysis

**Figure 4:**
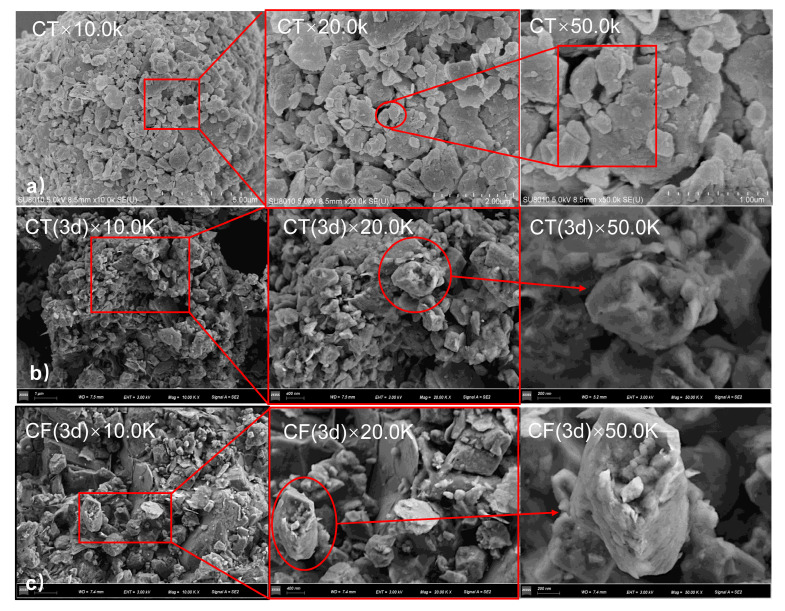
(**a**): SEM images of CT before sorption; (**b**): SEM images of CT with a sorption time of 3 d; (**c**): SEM images of CF with a sorption time of 3 d.

The surface micromorphology of the claystone samples before and after sorption observed by SEM is shown in Figure 4. The claystone sample before sorption (Figure 4a) had a laminated clastic structure with well-defined angles. Most of the fragments were approximately 1~5 μm in size and were stacked into clumps. The surface was loose and rough, containing multiple pore channels and irregular pore structures. Pore sizes ranged from 0.1 to 0.5 μm, which could provide good sites for Sr sorption.

A comparison of the microscopic sample morphology of claystone before and after Sr sorption shows that the sorption caused significant changes in the sample surface. After the sorption of Sr on CT, lamellar structures of different sizes and shapes were no longer obvious, and the complexity and roughness of the surface structure increased. In Figure 4b, some fragmented block structures and particle structures of different sizes can be observed. The opening of pores and voids increases, and the number of microporous structures decreases. This is because the claystone dissolved and precipitated during the reaction with the Sr solution. Precipitation, adsorbed Sr, and associated minerals adhered to the surface of the claystone sample changed the microscopic morphology of the claystone.

Under the action of FeOOH colloids, the microscopic morphology of claystone after adsorbing Sr is similar to that without colloids. The original clastic loose stacking structure of the claystone was replaced by a block structure and particle structure with different particle sizes. It can be observed from Figure 4c that under the action of FeOOH colloids, there were channels with irregular shapes and large openings, and these kinds of channels and holes cannot become the space for Sr sorption on the surface of the claystone. This also confirms that the sorption of Sr by CT does not conform to intraparticle diffusion; that is, the sorption of Sr by claystone is specific, and some large holes cannot provide sorption sites for Sr. In addition, needle-like or cylindrical needle ferrite colloids are seen on the claystone surface [52], which verifies the previous speculation that FeOOH colloids can occupy the sorption sites on the claystone surface and reduce its sorption capacity.

#### 3.2.3. Mineral Species Analysis

**Figure 5:**
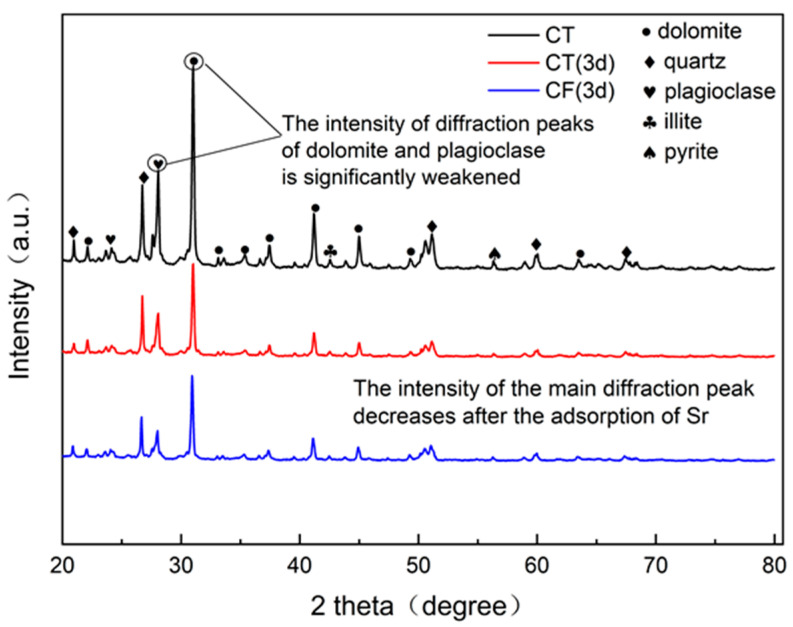
XRD spectra of CT and CF before and after sorption.

An X-ray diffractometer (DRON-4-07, Russia) was used to determine the characteristic changes in the mineral composition and unit cell structure of the claystone sample before and after sorption, and the results are shown in Figure 5. The comparison of the claystone XRD data with the standard powder diffraction file shows that the main minerals in the claystone samples are dolomite, quartz, plagioclase, illite, etc. Thus, the claystone contains a large amount of CaCO_3_, MgCO_3_, SiO_2_, and calcium aluminates. Comparing the XRD patterns before and after sorption shows that after the claystone adsorbed Sr, the positions of the main diffraction peaks moved in the direction of higher diffraction angles, and the intensities of the main diffraction peaks were weakened, among which the intensities of the diffraction peaks of plagioclase and dolomite were significantly weakened.

After Sr sorption by claystone, Ca and Mg ions in the minerals underwent cation exchange reactions with Sr [53,54]. The incorporation of Sr with smaller ionic radii in the crystalline cells of minerals changed the cell structure and decreased the lattice constant, so the intensity of the main diffraction peak decreased and shifted in the direction of higher angles. In conjunction with the EDS results of the claystone after Sr sorption, it can be seen that the atomic percentage content of Ca decreased by 4.48% and that of Mg decreased by 6.11%. Therefore, Sr can exchange ions with Ca and Mg in the claystone, and the reaction causes a significant weakening of the intensity of the diffraction peaks of dolomite and plagioclase.

#### 3.2.4. Functional Group Analysis

**Figure 6:**
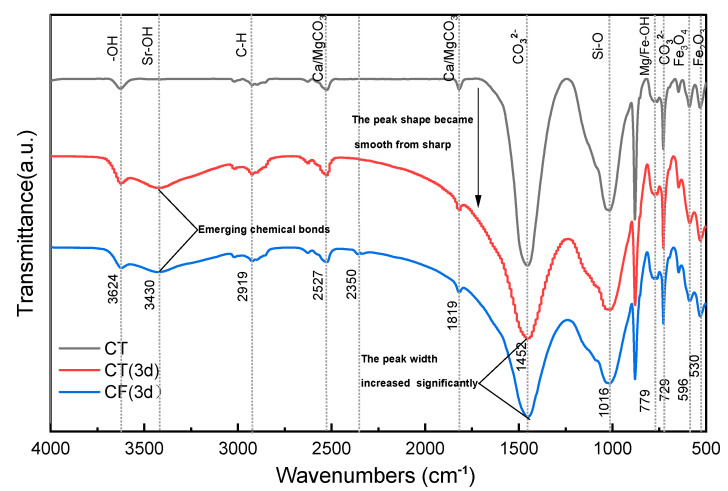
FTIR spectrum of claystone before and after the sorption of Sr.

Figure 6 shows the FTIR spectra of CT and CF. Based on the results of the tests and reports, the main functional groups in claystone samples are -OH, [CO_3_]^2−^, Si-O-Si, etc. [13,55,56]. In addition, the characteristic peaks of Fe_2_O_3_, Fe_3_O_4,_ carbonates, and metal hydroxides can also be detected in the spectrum.

The FTIR spectra of claystone before and after Sr sorption show that the peak shape of the FTIR curve of claystone changed greatly after Sr sorption. In the absence of colloids, the peak intensity of the sorption peak at 3624 cm^−1^ increased, but the displacement was not obvious. A new band appeared at 3430 cm^−1^, which had a weak peak intensity but a large peak width, indicating that the Sr in the solution formed a conjoining body with the free -OH in the claystone, and the degree of conjoining was high. In the process of Sr sorption by claystone, the chemical bond polarization was greatly increased and the vibration frequency was reduced due to intermolecular hydrogen bonding, so the peak intensity and peak width of the -OH sorption peak at 2700~2526.6 cm^−1^ were increased and slightly shifted to a lower band. The intensity of the sorption peak at 1819 cm^−1^ decreased, and the peak shape transformed from sharp to smooth. This is probably because when Mg/CaCO_3_ was replaced with Sr in solution, the C=O length increased, the force constant decreased due to the conjugation effect, and the density of the corresponding electron cloud decreased. The [CO_3_]^2^^−^ bending deformation vibration peak width at 1452 cm^−1^ increased more significantly after the sorption effect. This is mainly due to the bonding of Sr adsorbed on the claystone surface to form SrCO_3_, which reduced the vibrational frequency of this group. The peak width and peak intensity of the Mg/Fe-OH bond at 779 cm^−1^ increased after sorption. This is caused by the substitution reaction of the adsorbed Sr with Mg and Fe plasma, and the generated Sr-OH has a stronger vibration frequency and high sorption intensity. In the low band region, the FTIR spectra changed significantly after sorption. This can be explained by the change in the active groups of the claystone in this band due to sorption and the destruction of Fe_3_O_4_ and Fe_2_O_3_ with sorption.

Similar findings can be observed for the sorption of Sr by kaoslinite and magnesite [57] and minerals such as illite and goethite [58]. Sr could produce a complexation reaction with functional groups such as -OH and [CO_3_]^2−^ in claystone, adsorbing it to the claystone. At the same time, Sr can also have cation exchange reactions with Ca, Mg, etc. in the claystone. The reaction process can be expressed as follows:

(7) $$-\mathrm{OH}\;+{\;\mathrm{Sr}}^{2+}↔\;\mathrm{Sr}{\left(\mathrm{OH}\right)}^{+}$$

(8) $${\mathrm{CO}}_{3}^{2-}+{\;\mathrm{Sr}}^{2+}↔{\;\mathrm{SrCO}}_{3}\;\;\;\;$$

(9) $$\left(\mathrm{Ca},\mathrm{Mg}\right){\mathrm{CO}}_{3}+{\;\mathrm{Sr}}^{2+}↔{\mathrm{SrCO}}_{3}+{\left(\mathrm{Ca},\mathrm{Mg}\right)}^{2+}$$

(10) $$\left(\mathrm{Ca},\mathrm{Mg}\right){\mathrm{OH}}^{+}+{\;\mathrm{Sr}}^{2+}↔{\left(\mathrm{Ca},\mathrm{Mg}\right)}^{2+}+\mathrm{Sr}{\left(\mathrm{OH}\right)}^{+}$$

The FTIR spectrum changes under the colloidal effect of FeOOH are similar to those without the colloidal effect. The difference is that the claystone shows a new sorption peak at 2350 cm^−1^, which is presumed to be the result of the formation of Fe/Al-OH from the higher amounts of Fe and Al elements contained in the FeOOH colloid and the free -OH in the claystone. This finding can confirm the reduction of -OH functional groups on the surface of claystone under the action of the FeOOH colloid, which reduces the ability of claystone to adsorb Sr.

#### 3.2.5. Chemical Bond Analysis

**Figure 7:**
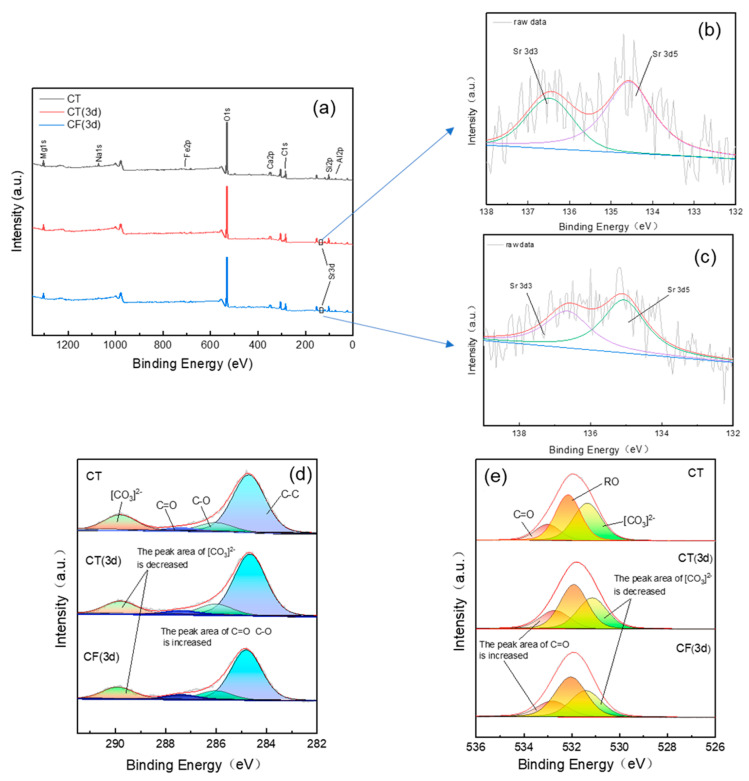
XPS spectra of claystone before and after sorption with CT and CF: (**a**) total survey scans; (**b**) Sr 3d spectra (CT 3d); (**c**) Sr 3d spectra (CF 3d); (**d**) C 1s spectra; (**e**) O 1s spectra.

The XPS spectra of CT and CF before and after Sr sorption (Figure 7) show that the claystone contains O, C, Ca, Mg, Na, Si, Fe, Al, and other elements.

Without the action of colloids, Sr appears in the full spectrum after 3 days of sorption, indicating that Sr has been adsorbed on the surface of the claystone, and its existence has changed the chemical environment of the surface of the claystone. The peak fitting of orbital C 1s in Figure 7c shows that the relative content of the [CO_3_]^2−^ functional group decreased from 0.18% (before sorption) to 0.14% (after sorption for 3 d), and the relative content of the C=O and C-O functional groups increased from 3.9% and 9.8% to 4.1% and 11.9%, respectively. Similarly, for the O 1s orbital, the relative content of the [CO_3_]^2−^ functional group also decreased, and the content of C=O and O-H increased. This indicates that functional groups such as [CO_3_]^2−^ and -OH on the claystone surface can react with Sr to form Sr-CO_3_ and Sr-OH association complexes [59,60], providing specific sites for Sr sorption on claystone.

The peak area of Sr in the full spectrum of CF is lower than that of CT, i.e., the Sr content on the surface of the claystone is lower, indicating that the ability of claystone to adsorb Sr is reduced under the action of the FeOOH colloid. It can be observed in Figure 7c,d that the decrease of [CO_3_]^2^^−^ in the CF spectrum and the increase of C=O and O-H functional groups are reduced compared with CT. As mentioned before, this is because FeOOH colloids having much higher surface area than CT may compete with CT to sorb Sr. And the sorption of FeOOH colloids on the claystone surface decreases the activity of the functional groups on the claystone surface, which decreases its sorption capacity.

## 4. Conclusions

The present study shows that FeOOH colloids had an inhibitory effect on Sr sorption by claystone, but their inhibitory effect was limited, and claystone still played a dominant role in the sorption process. The sorption of Sr by claystone was highly dependent on the solid content and pH value. The sorption increased with the solid content, and the claystone had a stronger sorption capacity for Sr under alkaline conditions. Moreover, the sorption process followed the PFO and PSO kinetic models, which are affected by physical diffusion and chemical sorption. According to the microscopic characterization analysis, the sorption of Sr by claystone is mainly by surface sorption, and the dolomite and plagioclase in claystone provide specific sorption sites for Sr sorption. The Ca and Mg in the minerals can react with Sr by cation replacement, and functional groups such as -OH and [CO3]^2^^−^ on the surface of the claystone can react with Sr by complexation. Therefore, the sorption behavior of Sr on the claystone surface consists mainly of ion-exchange reactions and complexation reactions. In addition, this study also found that FeOOH colloids attach to the claystone surface, reduce the sorption sites and the activity of functional groups on the claystone surface, and inhibit the sorption of Sr on claystone.

## Figures and Tables

**Table 1:** Results of sorption kinetic models of Sr sorption on CT and CF.

Sorption System	Sorption Kinetics	Parameter 1	Parameter 2	*R* ^2^
CT	Pseudo-first-order (PFO)	*q_e_* (mg·g^−1^) = 1.5615	*k_f_* (min^−1^) = 2.7433	0.9916
	Pseudo-second-order (PSO)	*q_e_* (mg·g^−1^) = 1.5629	*k_S_* (g∙mg^−1^∙min^−1^) = 11.1227	0.9904
	Elovich	*α* (g∙mg^−1^∙min^−1^) = 8.95 × 10^4^	*β* (g·mg^-1^) = 67.0384	0.9752
	Intraparticle Diffusion	*K_p_* (mg∙g^−1^∙min^−1/2^) = 0.0039	*C* (mg·g^-1^) = 1.3709	0.0366
CF	Pseudo-first-order (PFO)	*q_e_* (mg·g^−1^) = 1.5494	*k_f_* (min^−1^) = 1.9547	0.9832
	Pseudo-second-order (PSO)	*q_e_* (mg·g^−1^) = 1.5543	*k_S_* (g∙mg^−1^∙min^−1^) = 4.3983	0.9798
	Elovich	*α* (g∙mg^−1^∙min^−1^) = 3.71 × 10^4^	*β* (g·mg^−1^) = 67.3489	0.9563
	Intraparticle Diffusion	*K_p_* (mg∙g^−1^∙min^−1/2^) = 0.0038	*C* (mg·g^−1^) = 1.3536	0.0344

## Data Availability

Not applicable.

## Supplementary Materials

The following supporting information can be downloaded at: https://www.mdpi.com/article/10.3390/ijerph19169970/s1.
