# Supplementary material for: A Study of Sr Sorption Behavior in Claystone from a Candidate High-Level Radioactive Waste Geological Disposal Site under the Action of FeOOH Colloids
Source: Int J Environ Res Public Health. 2022 Aug 12;19(16):9970. doi: 10.3390/ijerph19169970 (PMC9408631; doi:10.3390/ijerph19169970)
Supplement: Supplementary file 1 [file ijerph-19-09970-s001.zip › ijerph-1860603-supplementary.pdf]

Supplementary Information

A Study of Sr Sorption Behavior in Claystone from A Candidate High-Level Radioactive Waste Geological Disposal Site: Under the Action of FeOOH Colloids

Jinsheng Wang [a,b], Weihai Cai[a,b] , Rui Zuo[a,b,*] and Can Du[c]

[a] College of Water Sciences, Beijing Normal University, Beijing 100875, China
[b] Engineering Research Center of Groundwater Pollution Control and Remediation, Ministry of Education, Beijing 100875, China
[c] Development and Research Center，China Geological Survey, Beijing 100037, China
* Correspondence: E-mail: zuo1101@163.com; Tel/Fax: +86-10-58802738.

S1. Materials and methods

S1.1 Materials and sample pretreatment

Claystone samples: CT samples were air-dried and refrigerated until they came to room temperature, crushed using a mortar, and passed through a 1.5-mm sieve.

Preparation of the FeOOH colloid: Approximately 101 g of $Fe(NO_3)_3·9H_2O$ was weighed and dissolved into a 250 mL solution. A sample of NaOH weighing 90 g was dissolved into a 500 mL solution. After preparation, the two solutions were mixed and the pH was adjusted to 12 with rapid stirring. The solution was diluted to 1 L and placed in a constant temperature water bath for 48 h. The temperature of the water bath was adjusted to 70 °C and not disturbed during this period. The resulting material was repeatedly washed until the supernatant was neutral and dried to produce the desired goethite particles. Five grams of the goethite powder were weighed and deionized water ultrapure water was again added. The solution was manually shaken repeatedly, and after there was no visible solid goethite in the container, it was placed into an ultrasonic dispersion cell to disperse for 1 h. The dispersed goethite suspension was kept for 12 h. After the solid component was precipitated, the solution was centrifuged at 6000 rpm for 30 min, and the supernatant was carefully extracted to prepare 1 L of the nanoscale FeOOH suspension.

S1.2 Methods

Batch tests: During the experiment, two different solutions were used: ① Sr solution (20 mg/L) and ② Sr solution (20 mg/L) + FeOOH colloid (10 mg/L). The solution volume was set to 250 mL, the temperature was 25 °C, and the adsorption time was 4320 min. The pH of the solutions was adjusted with 0.1 mol/L HCl and NaOH during the experiment. The different experimental conditions were set as shown in Table S2.

The collection and detection methods of the experimental samples were as follows: after the experiment, the supernatant was carefully extracted, one portion of the sample solution (S1) was retained, and one portion of the sample (S2) was passed through a 0.10 μm filter membrane and placed in a polyethylene container. The concentration of the Sr adsorption solution was determined by inductively coupled plasma atomic emission spectrometry (ICP-AES).

Since the particle size of all the prepared needle ferrite colloids was less than 0.45 μm and the dispersion of the solution was relatively stable over the experimental time, the possible components in sample I (S1) in this study included solid-phase media particles, colloid-mineral agglomerates, the Sr-colloid system, colloidal particles, and various ions formed by dissolution of media minerals; the colloidal material was filtered in sample II (S2).

S2 Characterization of the sample

S2.1 SEM measurements

The above samples were analyzed by scanning electron microscopy (SEM) (S-4800/EX-350, China), and the microscopic morphology of the claystone samples is shown in Figure S1(a). The claystone sample has a laminated clastic structure with well-defined angles, and most of the clastic particles are approximately 1~5 μm in size and stacked into clumps. The surface is loose and rough and porous, with irregular pore structures and pore sizes ranging from 0.5 to 5 μm. These pore channels provide good sites for Sr adsorption.

The microscopic morphology of the needle ferrite prepared in this study is shown in Figure S1(b). Under a 50.0 k magnification scanning electron microscope, it was observed that the FeOOH particles made in this study consisted of needle-like or cylindrical crystal structures of uniform length, morphology, and size. The crystal surface was smooth, the edges and corners were distinct, and the morphology was regular. The lengths were mostly in the range of 100~500 nm, and the needle-like or cylindrical crystal particles were aggregated into spherical or lumpy structures.

S2.2 FTIR measurements

Claystone samples and FeOOH samples were characterized by Fourier transform infrared spectrometry (FTIR) (NEXUS 670, USA), and the results are shown in Fig. S2. The absorption peaks in the CT characterization results are attributable as follows: the wavenumber at 3624 cm^{-1} is the interlayer, the peaks at 2527 and 1819 cm^{-1} are the weak absorption peaks of $Ca/MgCO_3$, the peaks at 1452 cm^{-1} and 731 cm^{-1} are attributed to the $[CO_3]^{2-}$ antisymmetric and bending vibration peaks in dolomite, respectively, and the peaks at 1016 cm^{-1} and 876 cm^{-1} are attributed to Si/Al-O antisymmetric and symmetric vibration peaks in quartz, respectively.

In the CF characterization results, iron oxide (≡Fe-OH) corresponds to multiple absorption peaks, among which 3121 cm^{-1} is the peak caused by the stretching vibration, 903 cm^{-1} is the peak caused by the in-plane bending vibration, and 798 cm^{-1} is the peak caused by the out-of-plane bending vibration. The absorption peak at 1787 cm^{-1} was caused by the stretching vibration of the C=O bond, the peak at 1647 cm^{-1} was caused by H-O-H angular vibration, and the functional group at 1134 cm^{-1} is Fe-OH, which is an important absorption peak of goethite. The stretching vibration frequencies of Fe-O in goethite are located at 608 cm^{-1} and 482 cm^{-1}.

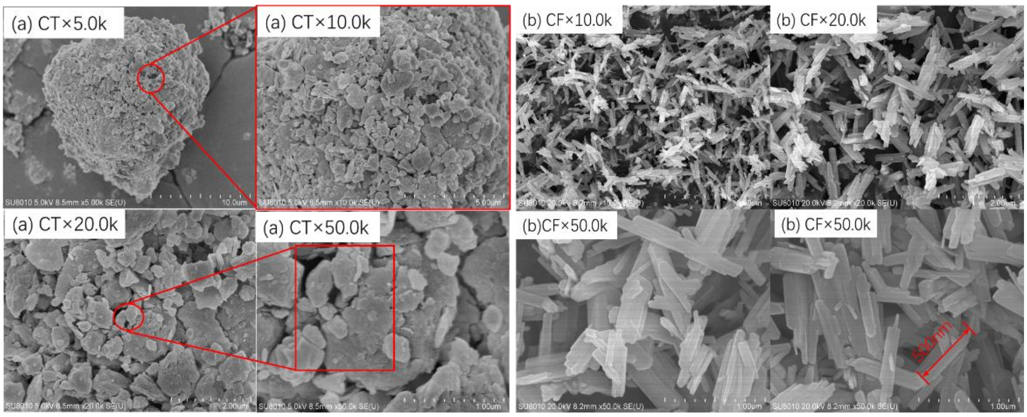

Figure S1. SEM images of CT and FC.

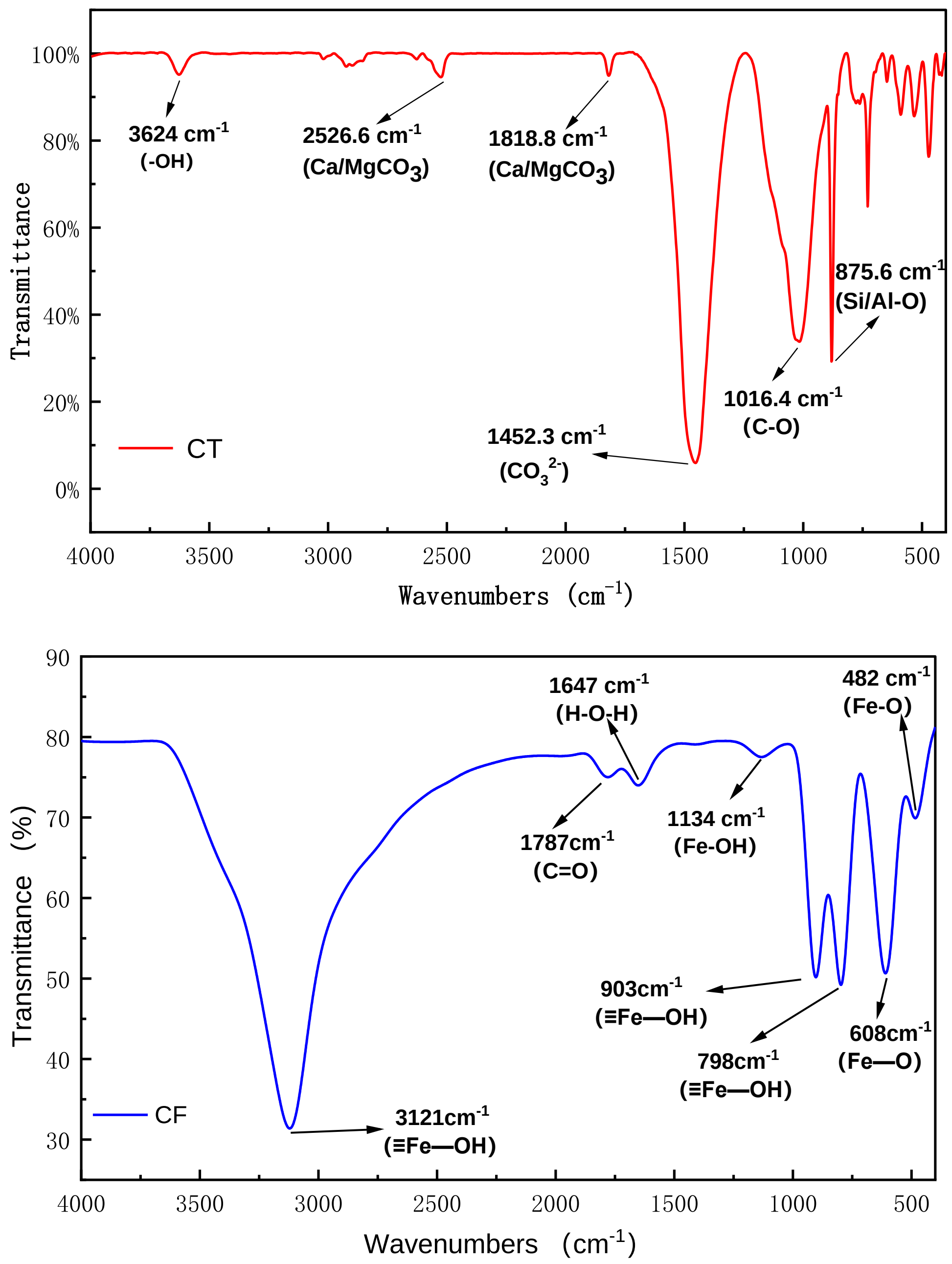

Figure S2. FT-IR spectra of CT and FC at wavenumbers 450-4000 $cm^{-1.}$

Table S1. Types and concentration of oxides in claystone samples determined by XRF.

Compound Name	Conc (%)	Compound Name	Conc (%)
SiO_2	36.528	CaO	24.727
MgO	14.113	Al_2O_3	10.001
Fe_2O_3	5.355	Na_2O	2.735
K_2O	2.699	SO_3	2.282
TiO_2	0.604	F	0.383
SrO	0.213	MnO	0.168
P_2O_5	0.088	Cl	0.039
Cr_2O_3	0.034	ZnO	0.018
Rb_2O	0.013		

Table S2. Controlled and varied factors of the experiments.

Varied / Factors	Colloid amounts (mL)	Contact time (min)	pH	Initial concentration of Sr (mg L^{-1})	Solid content (g L^{-1})	Temperature (°C)
Colloid amount (mL)	1, 5, 15, 25, 35, 50, 70	4320	7	20	10	25
Solid content(g L^{-1})	35.7	4320	7	20	1, 4, 10, 20, 40	25
pH	35.7	4320	3, 5, 7, 9, 11, 13	20	10	25
Contact time (min)	35.7	1, 5, 10, 30, 60, 90, 120, 240, 360, 720, 1440, 2880, 4320	7	20	20	25
